# Survey of integrative lumbar spinal stenosis treatment in Korean medicine doctors: preliminary data for clinical practice guidelines

**DOI:** 10.1186/s12906-017-1942-6

**Published:** 2017-08-29

**Authors:** Yoon Jae Lee, Joon-Shik Shin, Jinho Lee, Me-riong Kim, Yong-jun Ahn, Ye-sle Shin, Ki Byung Park, Byung-Cheul Shin, Myeong Soo Lee, Joo-Hee Kim, Jae-Heung Cho, In-Hyuk Ha

**Affiliations:** 1 Jaseng Spine and Joint Research Institute, Jaseng Medical Foundation, 858 Eonju-ro, Gangnam-gu, Seoul, Republic of Korea; 2 Division of Clinical Medicine, School of Korean Medicine, Pusan National University, Yangsan, Republic of Korea; 3 Medical Research Division, Korea Institute of Oriental Medicine, Daejeon, Republic of Korea; 4 Department of Korean Rehabilitation Medicine, College of Korean Medicine, Kyung Hee University, Seoul, Republic of Korea

**Keywords:** Spinal Stenosis, Health surveys, Complementary therapies, Integrative medicine

## Abstract

**Background:**

Considering that large variations exist amongst practitioners in lumbar disorder management and the significant costs that lumbar disorders incur, determining clinical practice patterns to provide preliminary data for standardization should be given higher priority. Lumbar spinal stenosis (LSS) is commonly treated using integrative non-surgical methods by Korean medicine doctors (KMDs) in Korea, and this is the first study to assess current Korean medicine practice trends for LSS.

**Methods:**

A survey on KMD diagnosis, treatment, prognosis and decision-making in LSS treatment was developed in a 3-step procedure of preliminary drafting, revision based on extramural expert opinion, and final editing. The survey was conducted at the internal conference of a spine-specialty Korean medicine hospital on January 25th, 2015.

**Results:**

The response rate was high at 79.19% (*n* = 118/149). Participants replied that they treated 7.3 ± 6.8 LSS patients/day using a multimodal treatment method consisting of acupuncture, pharmacopuncture, herbal medicine, Chuna manipulation, and electroacupuncture. Acupuncture mainly used Ashi points and MSAT, and pharmacopuncture mainly Shinbaro solution. The most frequently prescribed herbal medicine was Chungpa-jun, and the most commonly applied Chuna techniques were sidelying lumbar extension dysfunction correction technique, and prone lumbosacral joint distraction method. Radiological findings were mainly referred to for diagnosis, and clinical symptoms, age, radiological findings, and medical history were regarded to be important for prognosis. Participants replied that 7.8 ± 3.3 weeks were required for 50% reduction in pain, and 16.1 ± 7.7 weeks for 80% reduction.

**Conclusions:**

These results suggest that KMDs in Korea combine a conventional approach to LSS and a Korean medicine approach to low back pain for integration of empirical- and evidence-based diagnosis and treatment. The findings may contribute in bridging the divide between evidence and clinical practice guidelines for Korean medicine treatment of LSS and real-world clinical practice in future research.

**Electronic supplementary material:**

The online version of this article (10.1186/s12906-017-1942-6) contains supplementary material, which is available to authorized users.

## Background

Degenerative lumbar spinal stenosis (LSS) is a condition where the space for neurological and vascular structures is diminished secondary to degenerative changes of the lumbar spinal canal [1]. Prevalence estimates of relative LSS (≤12 mm) have been put at 23.6% in a community-based sample, and were highest in the 60–69 age group at 47.2% [2]. Quality of life is lower in LSS patients, which persists after surgery, stressing the importance of effective LSS treatment and management [3]. A surgical approach is commonly taken to LSS upon failure of conservative treatment, and the number of U.S. patients referred to lumbar fusion surgery has risen steeply [4]. Although the main body of evidence indicates that surgery is more effective than conservative treatment for certain outcomes (e.g. leg pain, disability) or at certain timepoints [5, 6], LSS patient samples and contents of nonsurgical treatment have been shown to be highly discrepant [7]. Also, a few studies have presented inconsistent outcomes with some reporting that surgical and conservative treatment beget similar results [8], while others have suggested that surgery prognosis may not be favorable in older patients [9, 10].

Conventional nonsurgical treatment generally refers to epidural injections, oral medication, and physical and manual therapy. However, current evidence for nonoperative LSS care is of low and very low quality with the exception of epidural injections [1], and is consequently not included in clinical practice recommendations or guidelines [7]. A recent systematic review concluded that level II evidence is available for long-term efficacy of caudal and interlaminar epidural injections, and level III for short-term improvement in transforaminal epidural injections [11].

A dual medical system of conventional medicine and Korean medicine is employed in Korea, and many patients seek Korean medicine for musculoskeletal disorder treatment. Various multimodal Korean medicine treatments including acupuncture, herbal medicine, Chuna manipulation, and pharmacopuncture are used for LSS treatment, and the treatment results of nonoperative Korean medicine combination therapy have been reported in a recent case series [12]. However, apart from a handful of retrospective studies and pilot trials which are mainly reports on specialized acupuncture techniques and multimodal Korean medicine treatments covering acupuncture, pharmacopuncture, Chuna manual therapy, herbal medicine, and education [12–14], current evidence on Korean medicine treatment for LSS is found largely lacking. Recently, more high-quality studies are being conducted [15], but evidence gaps still exist which conflict with its widespread clinical use in Korea, Moreover, further difficulties lie in performing current Korean medicine usage reports for LSS in that with the exception of acupuncture and electroacupuncture, many Korean medicine treatments are paid entirely out-of-pocket, and accurately assessing and monitoring current practice and usage patterns are therefore highly difficult. There are also no previous reports regarding the effects of or treatment duration needed to achieve improvement in integrative Korean medicine treatment for LSS. Moreover, integrative Korean medicine treatment for LSS differs from other conservative treatment modalities in numerous aspects, and these differences are also likely to be reflected in outcomes. Clinical studies observing real-world practice patterns have a higher probability to be successfully disseminated and implemented through clinical practice guidelines, and the current study was designed to outline Korean medicine practice patterns of LSS treatment to this aim. The survey was conducted in Korean medicine doctors (KMDs) specializing in spinal disorder treatment, the majority of whom were employed at a spine-specialty Korean medicine hospital. A specialty hospital refers to a hospital certified by the Minister of Health & Welfare to provide highly advanced, skilled medical care for a specific disease or specialty (Korea Medical law act 3, clause 5). This spine-specialty hospital was considered an appropriate study setting as it treats 90,000 spinal disorder patients annually.

This study holds significance as the first study to comprehensively report on clinical practice patterns and expert opinion of KMDs specializing in LSS treatment. The survey contents deal with how LSS is currently managed with Korean medicine along with details of diagnosis, treatment, prognosis and decision-making. These results are hoped to be of use to clinicians and researchers as preliminary data in designing clinical studies and establishing the foundation for clinical practice guidelines on LSS.

## Methods

### Questionnaire development

The questionnaire was developed in a 3-step procedure of preliminary drafting, revision based on extramural expert opinion, and final editing. Questionnaires on intervertebral disc displacement (IDD) and LSS were developed together, and the IDD survey results have been previously published in this journal. Further details of questionnaire development can be found in this article [16].

### Questionnaire distribution

The survey was conducted on January 25th, 2015 at a monthly internal conference held at a spine-specialty Korean medicine hospital. Sixty minutes were allocated for questionnaire completion, and inclusion was limited to KMDs currently engaging in outpatient practice. A researcher gave an explanation of the survey method to participants prior to filling out the questionnaire to minimize confusion. Absentees who had informed the board of their absence in advance were delivered the questionnaires through coworkers and asked to return the completed questionnaires by mail. This study was approved by the Institutional Review Board of Jaseng Hospital of Korean medicine in Korea (KNJSIRB2015–03), and informed consent was obtained without signatures to maintain anonymity. Respondents were informed of the objectives, questionnaire development procedure and survey completion method, and that personal information would be protected and that use of questionnaire results would be limited to academic means (See Additional file 1 for the final version of the questionnaire (translated from Korean)).

### Data entry

The data entry format was created by a designated statistician (KBP) using Microsoft Office Excel version 14.0 (Microsoft®, Redmond, WA, USA), and 2 trained researchers who did not participate in survey preparation or completion (JHL2, WKK) were assigned with electronic data entry. Any missing or ambiguous data were marked with color tabs, and the statistician (KBP), who did not participate in data entry, inspected all entries upon initial entry for verification and to check for any errors in conversion. Free responses of which the meaning was unclear from use of medical terminology or ambiguous terms were marked with different color tabs and delivered to a KMD (YSS) for resolution. Single answer selection items marked with multiple responses were processed as missing data.

### Data analysis

In descriptive statistics, continuous variables were presented as mean ± SD, and categorical data as frequency (%). Responses to Likert scales were quantified as continuous variables. The data allows for overlapping as most categorical data was collected with multiple response items, and participants were asked to rank items in the order of highest importance. PASW Statistics 18.0 (IBM Corporation, NY, USA) was used for all data analyses.

## Results

### Demographic characteristics of participant KMDs

A total 79.19% (*n* = 118/149) of the target population of KMDs listed to attend the conference responded, but 117 questionnaires were included in the final analysis as one respondent appeared to have misunderstood the whole questionnaire and was excluded from analysis. The KMDs participating in the survey were all male, and an average 38.6 ± 6.2 years. Average clinical experience was 12.1 ± 5.5 years, including 31 KMDs with ≥15 years’ experience. The highest level of education was Bachelor’s degree in 24.5%, Master’s in 35.8%, and Ph.D. in 39.8%. Regarding medical specialty, 76.4% were certified specialists, of whom 31.9% had specialized in Korean medicine rehabilitation, 28.7% in acupuncture and moxibustion, and 25.5% in Korean internal medicine. Korean medicine rehabilitation, and acupuncture and moxibustion are the two departments that treat the most spinal disorder patients in Korean medicine, and it is worth note that the majority of survey respondents came under those categories (Table 1).

**Table 1:** Demographics and clinical practice patterns of participating Korean medicine doctors

Factors	mean ± SD/n (%)
Age (years)	38.6 ± 6.2
30–39	85 (69.7)
40–49	27 (22.1)
≥ 50	10 (8.2)
Gender	
Male	123 (100)
Female	0 (0)
Clinical experience (years)	12.1 ± 5.5
5 ≤ <10	44
10 ≤ <15	48
15 ≤ <20	19
≥ 20	12
Level of healthcare facility of currently employed institution^a^	
Primary	39 (32)
Secondary	83 (68)
Highest academic degree	
Bachelor’s	30 (24.4)
Master’s	44 (35.8)
Ph. D.	49 (39.8)
Specialist training	
Yes (specialist)	94 (76.4)
No	29 (23.6)
Name of academic society in specialty for specialist training (if applicable)	
The Society of Korean Medicine Rehabilitation	30 (31.9)
Korean Acupuncture and Moxibustion Medicine Society	27 (28.7)
The Society of Internal Korean Medicine	24 (25.5)
Other	13 (13.8)

^a^Primary medical institutions operate <30 beds for inpatient care

Secondary medical institutions operate 30≤ and <500 beds for inpatient care, and at least 4 outpatient departments including medical specialties

### Clinical practice patterns of LSS

Surveyees replied that they treated 7.3 ± 6.8 LSS patients/day, and treatment usage rates were 96.7% for acupuncture, 94.3% for pharmacopuncture, 93.5% for herbal medicine, and 91.1% for Chuna manipulation, respectively, showing that in most cases an integrative multimodal treatment of acupuncture, pharmacopuncture, herbal medicine, and Chuna manipulation was administered. KMDs anticipated that 7.8 ± 3.3 weeks of treatment were needed for 50% decrease in pain, and 16.1 ± 7.7 weeks for 80% decrease (Table 2).

**Table 2:** Clinical practice patterns of LSS

Usage rate of treatment (multiple responses allowed) ( n (%))	
Acupuncture	119 (96.7)
Pharmacopuncture	116 (94.3)
Herbal medicine	115 (93.5)
Chuna manipulation	112 (91.1)
Cupping	104 (84.6)
Bee venom pharmacopuncture	96 (78.0)
Moxibustion	3 (2.4)
Clinical practice and time-to-effect (mean ± SD)	
Number of LSS outpatients consultations/day	7.3 ± 6.8
Number of treatment visits/week	1.9 ± 0.3
Average length of treatment required for 50% pain decrease (weeks)	7.8 ± 3.3
Average length of treatment required for 80% pain decrease (weeks)	16.1 ± 7.7

*LSS* lumbar spinal stenosis

Regarding the effects of individual interventions, bee venom pharmacopuncture was considered most significant in the short-term (≤8 weeks), followed by herbal medicine, pharmacopuncture, acupuncture, and Chuna manipulation, and herbal medicine was perceived to be the most significant in the long-term (1 year), followed by bee venom pharmacopuncture, pharmacopuncture, acupuncture, and Chuna manipulation (Table 3), indicating that KMDs regarded herbal medicine to be of greater importance in the long-term.

**Table 3:** Influence by factor in prognosis determination and importance of individual interventions for LSS

Treatment methods	Short term (8 weeks) importance	Long term (1 year) importance
	mean ± SD	mean ± SD
Herbal medicine	6.0 ± 1.1	6.6 ± 0.7
Bee venom pharmacopuncture	6.1 ± 1.0	5.7 ± 1.2
Pharmacopuncture	6.0 ± 0.9	5.7 ± 1.1
Acupuncture	5.8 ± 1.0	5.6 ± 1.2
Chuna manipulation	5.4 ± 1.2	5.4 ± 1.3
Cupping	4.5 ± 1.5	4.1 ± 1.5
Moxibustion	4.0 ± 1.6	4.3 ± 1.7

*LSS* lumbar spinal stenosis

### Diagnosis and prognosis determination of LSS

MRIs, X-rays, CTs, C-reactive protein (CRP), and digital infrared thermal imaging (DITI) were most frequently referred to for LSS diagnosis, and MRIs were reported to be used 98.4%, X-rays 94.3%, CTs 66.7%, CRP 9.8%, and DITIs 6.5%, revealing a strong preference for radiological findings. The factors rated to be most important in MRI readings were: the degree of nerve compression, spinal canal diameter, correlation between the level of disc dysfunction on MRI and clinical symptoms, and the degree of vertebral body or joint degeneration. DITI is a medical device that measures body temperature using digital infrared cameras, and is used as a means to quantify thermal asymmetry in low back pain (LBP) and sciatica [17]. The most frequently used physical examinations were the straight leg raise (SLR), followed by manual muscle testing, sensory testing, and heel walk/toe walk (Table 4). The most influential factors in LSS prognosis were regarded to be clinical symptoms, age, radiological findings, medical history, and patient perception of and attitude towards LSS (Table 5).

**Table 4:** Diagnostic tools used for LSS and Korean medicine syndrome differentiation

Factors		n (%)
Tests	Magnetic resonance imaging (MRI)^a^	121 (98.4)
	X-ray	116 (94.3)
	Computed tomography (CT)	82 (66.7)
	C-reactive protein (CRP)	12 (9.8)
	Digital infrared thermal imaging (DITI)	8 (6.5)
	Electromyogram	7 (5.7)
	Erythrocyte sedimentation rate (ESR)	7 (5.7)
Main points of consideration in MRI reading	Degree of nerve compression^a^	91 (74)
	Diameter/area of spinal canal	79 (64.2)
	Correlations between level of dysfunctional disc on MRI and clinical symptoms	66 (53.7)
	Degree of degeneration of vertebral body and/or joints (spondylosis)	46 (37.4)
	Number and level of dysfunctional discs (e.g. L1/2 vs. L5/S1)	30 (24.4)
	Degree of intervertebral disc displacement	28 (22.8)
	Degree of intervertebral disc degeneration	16 (13)
	Vertebral alignment	13 (10.6)
Physical examination	Straight leg raise test (SLR)^a^	94 (76.4)
	Manual muscle testing (MMT)	79 (64.2)
	Sensory testing	53 (43.1)
	Heel walk/toe walk	48 (39)
	Valsalva test	14 (11.4)
	Well leg raise test	14 (11.4)
	Milgram’s test	11 (8.9)
	Laseque sign	9 (7.3)
	Deep tendon reflex	9 (7.3)
	Bragard test	8 (6.5)
Korean medicine syndrome differentiation theory	Qi and Blood diagnosis (氣血辨證)^a^	83 (67.5)
	Eight principle pattern identification (八綱辨證)	82 (66.7)
	Meridian system diagnosis (經絡辨證)	82 (66.7)
	Organ system diagnosis (臟腑辨證)	62 (50.4)
	Six meridian diagnosis (六經辯證)	24 (19.5)
	Sasang constitutional medicine diagnosis (四象體質辨證)	21 (17.1)
	Defensive Qi and nutrient Blood diagnosis (衛氣營血辨證)	7 (5.7)
10 Types of LBP from ‘Dongeuibogam (東醫寶鑑)’	LBP from Kidney deficiency (腎虛腰痛)	108 (87.8)
	LBP from Cold pathogen (寒腰痛)	29 (23.6)
	LBP from Dampness pathogen (濕腰痛)	24 (19.5)
	LBP from Phlegm (痰飮腰痛)	20 (16.3)
	LBP from Blood stagnation (瘀血腰痛)	18 (14.6)
	LBP from Wind pathogen (風腰痛)	11 (8.9)
	LBP from Qi (氣腰痛)	5 (4.1)
	LBP from Dampness-Heat pathogen (濕熱腰通)	4 (3.3)
	LBP from contusion (挫閃腰痛)	2 (1.6)
	LBP from retention of food (食積腰痛)	2 (1.6)

*LSS* lumbar spinal stenosis, *MRI* magnetic resonance imaging, *LBP* low back pain

^a^Factor most frequently ranked 1st

**Table 5:** Prognostic factors regarding LSS

Prognostic factors	Importance
	mean ± SD
Clinical symptoms^a^	6.2 ± 1.0
Age	6.1 ± 1.1
Radiological findings	5.9 ± 1.1
Past history (e.g. surgery, trauma)	5.8 ± 1.2
Patient attitude toward disorder	5.6 ± 1.2
Time elapsed since onset and cause of onset	5.5 ± 1.3
Personality and other psychological factors (e.g. depression, anxiety)	5.3 ± 1.1
Physical examination	4.8 ± 1.4
Comorbidities	4.8 ± 1.3
Korean Medicine syndrome differentiation	4.3 ± 1.5

*LSS* lumbar spinal stenosis

^a^Factor most frequently ranked 1st

(Importance: 1 = not at all important, 2 = unimportant, 3 = somewhat unimportant, 4 = neither important nor unimportant, 5 = somewhat important, 6 = important, 7 = very important)

### Intervention usage for LSS

Reported use of herbal medicines in most frequently prescribed order was Chungpa-jun, Yookmijihwang-tang, Dokhwalgisaeng-tang, Hwalhyeoljitong-tang, and Ojeok-san. Even after due consideration of multiple responses, Chungpa-jun was prescribed predominantly at 99.2%. In Chuna manipulation, sidelying lumbar extension dysfunction correction technique, prone lumbosacral joint distraction method, spine flexion distraction method: flexion shift technique, prone posteriorly rotated ilium/sidebent sacrum correction technique, sidelying lumbar ‘pitch and roll’ distraction method, prone leg raise ilium correction technique, and prone anteriorly rotated ilium correction technique were the most commonly applied techniques. Usage of these 7 techniques was all within the range of 25.2% to 36.6%, implying that various techniques are used concomitantly according to primary dysfunction and patient condition. Ashi points, Motion Style Acupuncture Treatment (MSAT), and symptomatic needling were frequently used in acupuncture, and Shinbaro2 pharmacopuncture was most frequently used (69.9%) of pharmacopuncture types, followed by Shinbaro1 (69.1%), and Shinbaro3 (48.8%). Acupoints commonly used in manual acupuncture were Hyeopcheok (Huatuo Jiaji, EXB2), GB30 (環跳), Ashi, BL23 (腎兪), and BL40 (委中), and those used for pharmacopuncture were Hyeopcheok (Huatuo Jiaji, EXB2), Ashi, BL23 (腎兪), and GB30 (環跳) (Table 6).

**Table 6:** Commonly used Korean medicine treatments for LSS

Factors		n (%)
Herbal medicine	Chungpa-jun^a^	122 (99.2)
	Yookmijihwang-tang (六味地黃湯)	57 (46.3)
	Dokhwalgisaeng-tang (獨活寄生湯)	43 (35)
	Hwalhyeoljitong-tang (活血止痛湯)	30 (24.4)
	Ojeok-san (五積散)	30 (24.4)
	Shingi-hwan (腎氣丸)	19 (15.4)
	Danggwisoo-san (當歸鬚散)	13 (10.6)
	Jakyagkamcho-tang (芍藥甘草湯)	13 (10.6)
	Bojoongikgi-tang (補中益氣湯)	11 (8.9)
Chuna manipulation	Sidelying lumbar extension dysfunction correction technique	45 (36.6)
	Prone lumbosacral joint distraction method	41 (33.3)
	Spine flexion distraction method: Flexion shift technique	41 (33.3)
	Prone posteriorly rotated ilium/sidebent sacrum correction technique	36 (29.3)
	Sidelying lumbar ‘pitch and roll’ distraction method	35 (28.5)
	Prone leg raise ilium correction technique	32 (26)
	Prone anteriorly rotated ilium correction technique	31 (25.2)
	Prone sacrum sidebent rotation dysfunction correction technique	23 (18.7)
	Spine flexion distraction method: Extension technique	18 (14.6)
	Sidelying lumbar flexion dysfunction correction technique	13 (10.6)
Style of acupuncture	Ashi points	113 (91.9)
	Motion Style Acupuncture Treatment (MSAT)^a^	102 (82.9)
	Acupoints associated with symptoms (acupoints relating to specific disorder/syndromes)	91 (74)
	Dong-Si Acupuncture	14 (11.4)
Pharmacopuncture	Shinbaro 2^a^	86 (69.9)
	Shinbaro 1	85 (69.1)
	Shinbaro 3	60 (48.8)
	Joongseongouhyul (中性瘀血)	32 (26)
	Hwangryunhaedok (黃蓮解毒)	28 (22.8)
	Muscle relaxation	12 (9.8)
	Anti-inflammation	11 (8.9)
	Scolopendra	10 (8.1)
Acupoints used for acupuncture	Hyeopcheok (Huatuo Jiaji, EXB2) points	86 (69.9)
	GB30 (環跳)	60 (48.8)
	Ashi points	49 (39.8)
	BL23 (腎兪)	42 (34.1)
	BL40 (委中)	30 (24.4)
	BL25 (大腸兪)	23 (18.7)
Acupoints used for pharmacopuncture	Hyeopcheok (Huatuo Jiaji, EXB2) points	94 (76.4)
	Ashi points	43 (35.0)
	BL23 (腎兪)	35 (28.5)
	GB30 (環跳)	24 (19.5)
	BL25 (大腸兪)	21 (17.1)
	BL40 (委中)	4 (3.3)

*LSS* lumbar spinal stenosis

^a^Factor most frequently ranked 1st

### Acupuncture and pharmacopuncture usage for LSS

Items on acupuncture and pharmacopuncture usage are presented following the revised standards for reporting interventions in clinical trials of acupuncture (STRICTA) [18]. The rationale for acupuncture use was generally based on anatomical structures likely to cause symptoms, tender points, and pathological spine levels confirmed by imaging, and an average 12.5 ± 5.1 needles were inserted to a depth of 3.2 ± 1.4 cm per patient per session. Manual stimulation during acupuncture needle retention was used to the aim of de-qi (得氣) and muscle twitch response, and acupoint stimulation mainly used lifting and thrusting (提揷), holding and twisting (捻轉), and MSAT. Electroacupuncture use was reported at 91.3 ± 19.9%, showing a high usage rate, and average needle retention time was 14.2 ± 2.0 min.

Regarding the rationale for pharmacopuncture use, physical stimulation of the solution (100%), the chemical effect of the solution (97.6%), and the acupuncture effect of the pharmacopuncture needle (93.5%) were perceived to contribute to treatment effect. An average 3.1–6.1 acupoints were stimulated with a total 1.3–3.5cm^3^ of pharmacopuncture solution per patient per session (Table 7).

**Table 7:** Acupuncture and pharmacopuncture treatment used for LSS: data compiled and structured according to STRICTA standards

STRICTA checklist items		Acupuncture			Pharmacopuncture	
Acupuncture rationale	1a) Style of acupuncture	Refer to Table 4.		1a) Type of pharmacopuncture	Refer to Table 4.	
	1b) Reasoning for treatment provided	Anatomical structure that is probable cause of symptoms (e.g. shortened quadratus lumborum, shortened psoas muscles)^a^	85 (69.1)	1b) Reasoning for treatment provided	Physical stimulation of solution (i.e. irrigation of inflammation area, desensitization effect brought on through pain on injection)	123 (100)
		Tender points, trigger points, or other points that evoke a painful response on palpation	70 (56.9)			
		Pathological spine level as confirmed through imaging (e.g. level of disc herniation)	63 (51.2)		Chemical efficacy of solution (i.e. pharmaceutical effect of major componants)^a^	120 (97.6)
		Effective acupoints as observed through clinical experience	47 (38.2)			
		Ashi points (site of pain)	44 (35.8)		Acupuncture effects of pharmacopuncture needle (i.e. effect of pharmacopuncture needle itself)	115 (93.5)
		Acupoints following Korean medicine principle (e.g. GB30, BL40, BL57)	34 (27.6)			
		Academic knowledge from research articles, clinical practice guidelines	14 (11.4)		Placebo effect (i.e. effect from patient expectation)	7 (5.7)
		Knowledge from formal education	12 (9.8)			
Details of needling	2a) Number of needle insertions per patient per session		12.5 ± 5.1	2a) Number of acupoint injections per patient per session (range)		3.1 ~ 6.1
				2a) Amount of pharmacopuncture solution injected per session (range, cc)		1.3 ~ 3.5
	2b) Names of acupoints used	Refer to Table 4.		2b) Names of acupoints used	Refer to Table 4.	
	2c) Depth of insertion (cm)		3.2 ± 1.4	2c) Depth of insertion (range, cm)		1.7 ~ 3.7
	2d) Responses sought	De qi sensation	5.5 ± 1.4			
		Muscle twitch response	5.0 ± 1.4			
	2e) Needle stimulation	Motion Style Acupuncture Treatment (MSAT)	51 (41.5)			
		Lifting and thrusting (提揷)	64 (52.0)			
		Holding and twisting (捻轉)	62 (50.4)			
		Percentage of patients receiving electroacupuncture (%)	91.3 ± 19.9			
	2f) Needle retention time (minutes)		14.2 ± 2.0			
	2g) Needle type	Diameter of needle (mm)	0.3 ± 0.04			
Treatment regimen	3a) Number of sessions	Refer to Table 1.		3a) Number of sessions	Refer to Table 1.	
	3b) Frequency of treatment sessions (sessions/week)		1.9 ± 0.4	3b) Frequency of treatment sessions (sessions/week)		1.9 ± 0.4
	3b) Duration of treatment sessions (minutes)		18.7 ± 11.7	3b) Duration of treatment sessions (range, minutes)		2.3 ~ 4.3
Other components of treatment	4a) Other interventions administered	Refer to Table 1.		4a) Other interventions administered	Refer to Table 1.	
Practitioner background	5) Description of participating acupuncturists	Refer to Table 1.		5) Description of participating acupuncturists	Refer to Table 1.	

*LSS* lumbar spinal stenosis, *STRICTA* standards for reporting interventions in clinical trials of acupuncture

^a^Factor most frequently ranked 1st

### Safety

While respondents were asked to subjectively grade safety levels of individual treatment in the survey, data was not collected in reference to disorder type. The survey results of safety related items are specified in the previous publication on IDD [17].

## Discussion

Numerous RCTs on the effect of acupuncture and pharmacopuncture treatment for chronic LBP have been published [19–22]. However, as multimodal treatments including various interventions such as acupuncture, pharmacopuncture, and herbal medicine are widely applied in actual practice, the clinical relevance of efficacy studies on single interventions is difficult to gauge. Also, while several studies report the effect of nonoperative integrative Korean medicine treatment for lumbar IDD [23–25], there is limited research supporting use of such corresponding treatments for LSS. A meta-analysis on acupuncture for LSS found no conclusive evidence on effectiveness and safety due to high or uncertain risk of bias [26], and though a retrospective case series on integrative Korean medicine care for LSS has been reported [12], there is a dearth of well-designed prospective studies and RCTs.

This survey study investigated use of integrative Korean medicine treatment for LSS and collected expert opinion in clinicians specializing in Korean medicine treatment of LSS. Detailed information on treatment contents and minimum duration of treatment required for favorable outcomes were collected to integrate real-world LSS practice into clinical study design and to provide preliminary data for future clinical practice guidelines. Many of the hospital sites included in this study were spine-specialty Korean medicine hospitals, and the hospital that the surveyees were affiliated at is composed of 19 hospital sites and clinics in Korea and 6 branches in the U.S. as of 2017, treating over 900,000 spinal and joint disorder cases a year with an integrative Korean medicine program mainly consisting of acupuncture, Chuna manipulation, herbal medicine, and pharmacopuncture [27]. The integrative treatment method of IDD used at this hospital has been introduced in several previous publications [23, 25], and the treatment outcomes for LBP have also been shown to be favorable [27–29]. Questionnaire contents were prepared through preliminary drafting, revision, and editing. KMDs with clinical experience participated in questionnaire compilation drawing from various references, and aimed to increase external validity and credibility through extramural expert review [16].

Respondents replied that they treated 7.3 ± 6.8 LSS patients/day on average, and that they expected 7.8 ± 6.8 treatment sessions to be needed for 50% pain reduction. Diagnostic imaging was considered most important in diagnosis, and MRIs, X-rays, and CTs were the main imaging tools of choice. Integrative care consisting of acupuncture, herbal medicine, Chuna manipulation, and pharmacopuncture was commonly administered, of which acupuncture mainly used Ashi points and MSAT, and pharmacopuncture mainly Shinbaro solution. The most frequently prescribed herbal medicine was Chungpa-jun, and the most commonly applied Chuna techniques were sidelying lumbar extension dysfunction correction technique, prone lumbosacral joint distraction method, spine flexion distraction method: flexion shift technique, and prone posteriorly rotated ilium/sidebent sacrum correction technique. Usage rates of frequently employed Chuna techniques were similar, suggesting that a range of combined variations are used with respect to patient condition and examination results. KMDs considered LSS to be most similar to LBP from Kidney deficiency of the 10 types of LBP from ‘Dongeuibogam (東醫寶鑑)’, and in prognosis determination, clinical symptoms, age, radiological findings, and medical history were regarded to be the most significant factors.

Some points worth notice include that MSAT, whose effects on acute LBP have been reported in a previous RCT [29], was widely applied to LSS patients, and that Shinbaro pharmacopuncture, whose effectiveness has been clinically proven mainly in IDD populations [30], was likewise applied to LSS, suggesting that it was used under the assumption that it would display similar mechanisms and outcomes in LSS treatment.

Also, as the original survey was devised to address IDD and LSS together, comparison of current survey results with previous IDD findings holds clinical significance. Symptoms, followed by age, radiological findings, and past history were viewed to hold the most weight in LSS prognosis, showing that KMDs considered patient age to be a more important factor in LSS compared to IDD. While frequently used diagnostic tools were comparable in IDD and LSS with MRI, X-ray, CT, and CRP most referred to, the main focal points in MRI readings were contrasting: Whereas KMDs tended to focus on the degree of intervertebral disc displacement, the degree of nerve compression, and correlation between the level of disc displacement on MRI and clinical symptoms in IDD patients, the degree of nerve compression and diameter/area of the spinal canal were purported to be the most relevant in LSS. Physical examinations of interest were also dissimilar with SLR, manual muscle testing, and sensory testing frequently used in LSS, suggesting heightened importance of motor strength and sensory evaluation in treatment of LSS patients compared to IDD. In drawing correlations between LSS and the 10 types of LBP from ‘Dongeuibogam (東醫寶鑑)’, LSS was considered most compatible with LBP from Kidney deficiency and LBP from Cold pathogen (寒腰痛), which shows marked difference from that of IDD, where it was perceived to be most similar to LBP from Blood stagnation (瘀血腰痛), Phlegm (痰飮腰痛), and contusion (挫閃腰痛). Difference in differential pattern diagnosis also influenced herbal medicine prescription: While Chungpa-jun was regarded to be most significant in both IDD and LSS, the second and third most important prescriptions were highly disparate. A total 46.3% selected Yookmijihwang-tang in LSS, making it the second choice, and it was followed by Dokhwalgisaeng-tang, which reflects perception of LSS as a condition resembling ‘LBP from Kidney deficiency’.

To summarize, KMDs associated LSS more with LBP from Kidney deficiency and LBP from Cold pathogen (寒腰痛), gave more consideration to such factors as age and history than in IDD, and Yookmijihwang-tang, a prescription used to treat LBP from Kidney deficiency was more frequently prescribed for LSS. Furthermore, anatomical knowledge and conventional diagnostic methods were given high priority in treating LSS patients which is reflected in the focus on such structural aspects as the diameter/area of the spinal canal in diagnostic testing in light of pathological characteristics, and in higher use of motor function and sensory testing. Still, Korean medicine treatment for LSS also shared several common factors with IDD, as can be observed in the similarly high usage rate of Chungpa-jun and contents of integrative treatment. This may be due in part to the Korean medicine approach to both LSS and IDD in the wider category of LBP. In conclusion, though KMDs refer to Korean medicine diagnosis, this population had a high preference for Chungpa-jun, whose in vivo and in vitro anti-inflammatory, neuroprotective, and cartilage-protective effects and clinical effect in IDD and arthritis have been proven [25, 31–34], and which may be read in line with the emphasis placed on clinical symptoms, age, and radiological findings in LSS prognosis.

While it is general practice for KMDs to prescribe traditional Korean medicine treatments including herbal medicine based on individual constitution, overall condition, or syndrome differentiation rather than a primarily disease-focused approach [35], the majority of KMDs in this study showed high preference for Chungpa-jun, indicating otherwise. This difference is probably due to the awareness of respondents of the evidence for the anti-inflammatory, neuroprotective, and cartilage-protective effects of Chungpa-jun [25, 31–34], and it can be carefully inferred that the surveyed KMDs prescribe herbal medicine with a more evidence-based approach as opposed to an exclusively traditional, Korean medicine syndrome differentiation-based approach.. In addition, although these KMDs also employed a Korean medicine approach in diagnosis as evidenced by their evaluation of the main cause of LBP as from Kidney deficiency (腎虛腰痛), they were shown to incorporate it with a contemporary conventional medicine approach towards an integrative medicine model as exemplified in the common use of MRI readings for diagnosis and Chungpa-jun for treatment. Also, regarding terminology, there is a major trend across Korean medicine towards more standardized and consistent use of concurrent diagnostic terms such as IDD and LSS to promote wider understanding among patients, physicians, and researchers, as opposed to more comprehensive and traditional terms. These results show that KMDs in Korea incorporate a conventional approach to LSS with traditional Korean medicine perspectives of LBP making use of current evidence, anatomical knowledge and conventional diagnostic methods with Korean medicine treatment modalities for evidence-based integrative LSS diagnosis and treatment.

Limitations of this study include that as a survey study, it is susceptible to recall bias in contrast to clinical studies on patient populations, and treatment effects may have been overestimated in time-to-effect items as they do not consider for dissatisfied patients prematurely terminating treatment. Also, although this questionnaire was developed through extramural expert consultation and review, and the participation of various clinical specialists in an effort to increase external credibility, the questionnaire is still limited in that reliability and validity analyses were not conducted. In addition, as the survey was mainly conducted in KMDs working at spine-specialty Korean medicine hospitals and specializing in spinal disorders, the results cannot be taken to represent the whole KMD population. Especially as all survey respondents were male KMDs, the findings are limited in that they do not reflect the opinion of the 14.4% females of the total KMD demographic. In addition, while 69.7% of respondents were in their 30s, only an estimated 36.3% are in their 30s out of total KMDs, and these results cannot be taken to be representative of the general KMD population [36]. Still, the KMDs surveyed in our study responded that they treated 16.1 ± 7.2 IDD patients and 7.3 ± 6.8 LSS patients per day, suggesting that these findings depict current clinical practice patterns of physicians treating a sizable patient pool. The questionnaire was developed and outcomes were analyzed in tandem with extramural guidance, and the most significant strength of this study is that it is the first detailed report of Korean medicine practice patterns and Korean medicine expert opinion of LSS treatment. Future studies with revised questionnaires (reworked based on current findings) conducted in larger KMD populations are warranted for wider implication. Factors such as patient age and comorbidities may potentially influence decision making in LSS, and the authors would like to propose the use of patient case examples for more specific determination of treatment contents, duration and frequency in future survey studies. As the level of evidence is low in most treatments for LSS aside from surgery and the concern for surgical complications increases in older patient groups, the option of nonsurgical integrative Korean medicine treatment of LSS may hold more universal appeal. These study results imply that various Korean medicine treatments are widely applied for LSS. Further well-designed clinical studies on Korean medicine treatment efficacy and safety reflecting clinical practice patterns should contribute to implementation of evidence-based complementary and alternative medicine (CAM) treatment.

## Conclusions

This survey study consisted of the status report and expert opinion on Korean medicine treatment of LSS in physicians specializing in LSS treatment. The survey covered various aspects of contemporary Korean medicine clinical practice including treatment duration, diagnosis, details of acupuncture and pharmacopuncture treatment, herbal medicine, and prognosis determination. The results indicate that KMDs in Korea have combined a conventional approach to LSS using mainly conventional diagnostic devices with traditional Korean medicine treatment perspectives of LBP for a unique take on evidence-based integrative LSS diagnosis and treatment. These findings are expected to contribute to future clinical studies on Korean medicine treatment of LSS.

## Additional file

Additional file 1: Clinical practice of Korean medicine for lumbar spinal stenosis: A survey. The final questionnaire used for collection of data. (DOCX 88 kb)

## Abbreviations

CAM: Complementary and alternative medicine
CRP: C-reactive protein
DITI: Digital infrared thermal imaging
IDD: Intervertebral disc displacement
KMD: Korean medicine doctor
LBP: Low back pain
LSS: Lumbar spinal stenosis
MSAT: Motion style acupuncture treatment
SLR: Straight leg raise
STRICTA: Standards for reporting interventions in clinical trials of acupuncture

## References

[1] Kreiner DS, Shaffer WO, Baisden JL, Gilbert TJ, Summers JT, Toton JF (2013). An evidence-based clinical guideline for the diagnosis and treatment of degenerative lumbar spinal stenosis (update). Spine J.

[2] Kalichman L, Cole R, Kim DH, Li L, Suri P, Guermazi A (2009). Spinal stenosis prevalence and association with symptoms: the Framingham study. Spine J.

[3] Jansson KA, Nemeth G, Granath F, Jonsson B, Blomqvist P (2009). Health-related quality of life (EQ-5D) before and one year after surgery for lumbar spinal stenosis. J Bone Joint Surg Br.

[4] Deyo RA, Gray DT, Kreuter W, Mirza S, Martin BI (2005). United States trends in lumbar fusion surgery for degenerative conditions. Spine.

[5] Weinstein JN, Tosteson TD, Lurie JD, Tosteson AN, Blood E, Hanscom B (2008). Surgical versus nonsurgical therapy for lumbar spinal stenosis. N Engl J Med.

[6] Jacobs WC, Rubinstein SM, Koes B, van Tulder MW, Peul WC (2013). Evidence for surgery in degenerative lumbar spine disorders. Best Pract Res Clin Rheumatol.

[7] Ammendolia C, Stuber KJ, Rok E, Rampersaud R, Kennedy CA, Pennick V (2013). Nonoperative treatment for lumbar spinal stenosis with neurogenic claudication. Cochrane Db Syst Rev.

[8] Delitto A, Piva SR, Moore CG, Fritz JM, Wisniewski SR, Josbeno DA (2015). Surgery versus nonsurgical treatment of lumbar spinal stenosis: a randomized trial. Ann Intern Med.

[9] Ciol MA, Deyo RA, Howell E, Kreif S (1996). An assessment of surgery for spinal stenosis: time trends, geographic variations, complications, and reoperations. J Am Geriatr Soc.

[10] Deyo RA, Cherkin DC, Loeser JD, Bigos SJ, Ciol MA (1992). Morbidity and mortality in association with operations on the lumbar spine. The influence of age, diagnosis, and procedure. J Bone Joint Surg Am.

[11] Manchikanti L, Kaye AD, Manchikanti K, Boswell M, Pampati V, Hirsch J (2015). Efficacy of epidural injections in the treatment of lumbar central spinal stenosis: a systematic review. Anesth Pain Med.

[12] Kim K, Jeong Y, Youn Y, Choi J, Kim J, Chung W (2015). Nonoperative Korean medicine combination therapy for lumbar spinal Stenosis: a retrospective case-series study. Evid-Based Compl Alt..

[13] Kim KH, Kim YR, Baik SK, Noh SH, Kim DH, Lee SW (2016). Acupuncture for patients with lumbar spinal stenosis: a randomised pilot trial. Acupunct Med.

[14] Lee GM, Lee EY, Han JH, Cho KH, Kang SR, Yoon SH (2014). Effects of Wonli acupuncture procedure in patients with LSS: a clinical. Retrospective Study Evid-Based Compl Alt.

[15] Kim K, Shin K-M, Lee J-H, Seo B-N, Jung S-Y, Youn Y, et al. Nonsurgical Korean Integrative Treatments for Symptomatic Lumbar Spinal Stenosis: A Three-Armed Randomized Controlled Pilot Trial Protocol. Evid-Based Compl Alt. 2016;2016. 10.1155/2016/2913248 PMC4749804 26941823

[16] Shin YS, Shin JS, Lee J, Lee YJ, Kim MR, Ahn YJ (2015). A survey among Korea medicine doctors (KMDs) in Korea on patterns of integrative Korean medicine practice for lumbar intervertebral disc displacement: preliminary research for clinical practice guidelines. BMC Complem Altern Med.

[17] Uematsu S, Jankel WR, Edwin DH, Kim W, Kozikowski J, Rosenbaum A (1988). Quantification of thermal asymmetry: part 2: application in low-back pain and sciatica. J Neurosurg.

[18] MacPherson H, Altman DG, Hammerschlag R, Youping L, Taixiang W, White A (2010). Revised STandards for reporting interventions in clinical trials of acupuncture (STRICTA): extending the CONSORT statement. J Evid Based Med.

[19] Shin B-C, Kong JC, Park T-Y, Yang C-Y, Kang K-W, S-m C (2012). Bee venom acupuncture for chronic low back pain: a randomised, sham-controlled, triple-blind clinical trial. Eur J Integr Med..

[20] Molsberger AF, Mau J, Pawelec DB, Winkler J (2002). Does acupuncture improve the orthopedic management of chronic low back pain--a randomized, blinded, controlled trial with 3 months follow up. Pain.

[21] Zaringhalam J, Manaheji H, Rastqar A, Zaringhalam M (2010). Reduction of chronic non-specific low back pain: a randomised controlled clinical trial on acupuncture and baclofen. Chin Med.

[22] Vas J, Aranda JM, Modesto M, Benitez-Parejo N, Herrera A, Martinez-Barquin DM (2012). Acupuncture in patients with acute low back pain: a multicentre randomised controlled clinical trial. Pain.

[23] Shin J-S, Lee J, Kim M-r, Shin B-C, Lee MS, Ha I-H (2014). The long-term course of patients undergoing alternative and integrative therapy for lumbar disc herniation: 3-year results of a prospective observational study. BMJ Open.

[24] Yuan WA, Huang SR, Guo K, Sun WQ, Xi XB, Zhang MC (2013). Integrative TCM conservative therapy for low back pain due to lumbar disc herniation: a randomized controlled clinical trial. Evid-Based Compl Alt..

[25] Park JJ, Shin J, Choi Y, Youn Y, Lee S, Kwon S-R (2010). Integrative package for low back pain with leg pain in Korea: a prospective cohort study. Complement Ther Med..

[26] Kim KH, Kim T-H, Lee BR, Kim JK, Son DW, Lee SW (2013). Acupuncture for lumbar spinal stenosis: a systematic review and meta-analysis. Complement Ther Med.

[27] Robinson N, Liu J (2012). Oriental and traditional medicine–supporting the vision for integrated health. Eur J Integr Med..

[28] Stevens L, Duarte H, Park J (2007). Promising implications for integrative medicine for back pain: a profile of a Korean hospital. J Altern Comp Med.

[29] Shin J-S, Ha I-H, Lee J, Choi Y (2013). Kim M-r, park B-Y, et al. effects of motion style acupuncture treatment in acute low back pain patients with severe disability: a multicenter, randomized, controlled, comparative effectiveness trial. Pain.

[30] Jun B, Kim E, Kim D, Kim T, Kim J (2011). Effectiveness of ShinBaro pharmacopuncture on lumbar spinal herniated intervertebral disc: a randomized controlled trial. J Korea CHUNA Manual Med Spine Nerves.

[31] Chung H-J, Lee H-S, Shin J-S, Lee S-H, Park B-M, Youn Y-S (2010). Modulation of acute and chronic inflammatory processes by a traditional medicine preparation GCSB-5 both in vitro and in vivo animal models. J Ethnopharmacol.

[32] Kim T-H, Yoon S-J, Lee W-C, Kim J-K, Shin J, Lee S (2011). Protective effect of GCSB-5, an herbal preparation, against peripheral nerve injury in rats. J Ethnopharmacol.

[33] Kim J-K, Park S-W, Kang J-W, Kim Y-J, Lee SY, Shin J (2012). Effect of GCSB-5, a herbal formulation, on monosodium iodoacetate-induced osteoarthritis in rats. Evid-Based Compl Alt.

[34] Park Y-G, Ha C-W, Han C-D, Bin S-I, Kim H-C, Jung Y-B (2013). A prospective, randomized, double-blind, multicenter comparative study on the safety and efficacy of Celecoxib and GCSB-5, dried extracts of six herbs, for the treatment of osteoarthritis of knee joint. J Ethnopharmacol.

[35] Park T-Y, Lee JA, Cha MH, Kang B-K, Moon T-W, Choi T-Y (2012). The fundamental study for the standardization and objectification of pattern identification in traditional Korean medicine for stroke (SOPI-stroke): an overview of phase I. Eur J Integr Med.

[36] Employment Information Research Division of Korea Employment Information Service (2015). 2015 Korea occupational outlook. Korea employment information service.

